# A high-quality chromosome-level genome assembly of *Pelteobagrus vachelli* provides insights into its environmental adaptation and population history

**DOI:** 10.3389/fgene.2022.1050192

**Published:** 2022-11-14

**Authors:** Jie Li, Tao Wang, Wei Liu, Danqing Yin, Zhengqing Lai, Guosong Zhang, Kai Zhang, Jie Ji, Shaowu Yin

**Affiliations:** ^1^ College of Marine Science and Engineering, Jiangsu Province Engineering Research Center for Aquatic Animals Breeding and Green Efficient Aquacultural Technology, Nanjing Normal University, Nanjing, Jiangsu, China; ^2^ Co-Innovation Center for Marine Bio-Industry Technology of Jiangsu Province, Lianyungang, Jiangsu, China; ^3^ Institute of Fisheries Science of Nanjing, Nanjing, China; ^4^ School of Biomedical Sciences, Li Ka Shing Faculty of Medicine, University of Hong Kong, Pokfulam, Hong Kong SAR, China

**Keywords:** *Pelteobagrus vachelli*, genomics, chromosomal assembly, population history, environmental adaptation

## Abstract

*Pelteobagrus vachelli* is a freshwater fish with high economic value, but the lack of genome resources has severely restricted its industrial development and population conservation. Here, we constructed the first chromosome-level genome assembly of *P. vachelli* with a total length of approximately 662.13 Mb and a contig N50 was 14.02 Mb, and scaffolds covering 99.79% of the assembly were anchored to 26 chromosomes. Combining the comparative genome results and transcriptome data under environmental stress (high temperature, hypoxia and *Edwardsiella. ictaluri* infection), the MAPK signaling pathway, PI3K-Akt signaling pathway and apelin signaling pathway play an important role in environmental adaptation of *P. vachelli*, and these pathways were interconnected by the ErbB family and involved in cell proliferation, differentiation and apoptosis. Population evolution analysis showed that artificial interventions have affected wild populations of *P. vachelli*. This study provides a useful genomic information for the genetic breeding of *P. vachelli*, as well as references for further studies on fish biology and evolution.

## Highlights

1) The first high-quality genome of *P. vachelli* (contig N50 = 14.02 Mb)*.*
2) Cell proliferation, differentiation and apoptosis play an important role in environmental adaptation of *P. vachelli.*
3) The environmental adaptation pathways and candidate genes of *P. vachelli* were identified.
4) Artificial interventions have affected wild populations of *P. vachelli.*

## 1 Introduction

*Pelteobagrus vachelli* (Siluriformes, Bagridae) is an omnivorous freshwater fish that is mainly distributed in the Yangtze River and its tributaries (Li et al., 2015; Zhang et al., 2016a). This fish has been one of the top consumer choices in the domestic freshwater fish market because of the less intermuscular spines, high nutrition, taste, and tenderness (Zhang et al., 2016a). The high market value and consumer demand have promoted the rapid industrialization of *P. vachelli* farming. This rapid scaling up of the product has helped to meet the market demand however has come up with several problems.

The artificial breeding program and high-density farming have been associated with physiological stresses like high temperature, hypoxia, and bacterial-borne diseases in the *P. vachelli* population, impacting the fish’s health, quality and quantity of the production (Zhang et al., 2016a; Zhang et al., 2016c; Qin et al., 2018). The natural living environment of flowing water is responsible for the intolerance of *P. vachelli* to hypoxia. In fact, continuous rainy weather or high breeding density will cause hypoxia death in pond aquaculture and remarkable economic losses in the farmer industry (Zhang et al., 2017a). Water pollution caused by intensive farming will lead to an increase in pathogenic bacteria, causing the outbreak of various diseases. In addition, the high temperature caused by global warming further exacerbates hypoxia and disease outbreaks in aquaculture. With the recent development of sequencing technology, transcriptomics (Zhang et al., 2017b; Qin et al., 2017) and proteomics (Zhang et al., 2017a; Li et al., 2019) have been used to explore the mechanisms of hypoxic stress and innate immunity in *P. vachelli*; however, the accuracy of omics annotation cannot be guaranteed due to a lack of high-quality reference genomes.

With the rapid development of the industrial scale of *P. vachelli*, the brood stock on the market can no longer meet the demand for artificial breeding. Prior to 2019, remarkable amounts of *P. vachelli* were caught in the Yangtze River for seed production, which severely damaged and disturbed its wild population. Studies have shown that the genetic diversity of *P. vachelli* in the Yangtze River has decreased in recent years (Zheng et al., 2020). Population evolution analysis based on whole genome sequencing (WGS) can help us understand the evolutionary history and current status of its population to formulate corresponding protection measures (Hu et al., 2021).

In this study, we reported the first chromosome-scale genome assembly of *P. vachelli*. To the best of our knowledge, except for that of *Thamnaconus septentrionalis* (contig N50 = 22.46 Mb) (Bian et al., 2020), the genome of *P. vachelli* (contig N50 = 14.02 Mb) has the highest quality among published fish genomes. The comparative genome and population evolution analyses performed herein provided insights into environmental adaptation and population history. Accordingly, the findings of this study can be used as a genetic basis for future biological research on *P. vachelli*, ultimately providing valuable resources for genetic breeding and population conservation.

## 2 Materials and methods

### 2.1 Sample collection and genome sequencing

The XX genotype female Darkbarbel catfish, *P. vachelli*, was collected from Meishan City, Sichuan Province. After dissecting the fish, muscle tissue was obtained and flash-frozen in liquid nitrogen and stored at −80°C. High-quality genomic DNA was extracted from the muscle using a modified CTAB method (Cota-Sanchez et al., 2006). RNase A (0.1 ml 100 mg/L RNase, 37°C for 30–60 min) was used to remove RNA contaminants. The quality of the DNA was checked using Agilent 2100 Bioanalyzer (California, United States) and high integrity DNA molecules were measured using 1% agarose gel electrophoresis. Sequencing libraries were generated using VAHTS Universal DNA Library Prep Kit for MGI (Vazyme, Nanjing, China) following the manufacturer’s instructions. Index codes were used to cross-index the sequences and samples, that was, the DNA samples were fragmented by sonication and then end-polished, A-tailed, and ligated with the full-length adapter for MGI sequencing followed by PCR amplifcation. The resulting PCR products were purifed (AMPure XP system) and the sequence libraries were analyzed for size distribution by Agilent 2100 Bioanalyzer and quantifed using real-time PCR. A total of one 300–500 bp short-insert libraries and one 40 kb long-insert libraries were prepared. Then, the MGISEQ 2000 platform (BGI, Shenzhen, China) (Chen et al., 2019) and PacBio Sequel II (Menlo Park, CA, United States) (Rhoads and Au, 2015) were employed for whole-genome sequencing. Short reads generated from the MGI platform were used to estimate the genome size, the level of heterozygosity, and repeat content of the genome. Long reads from the PacBio platform were used for genome assembly.

### 2.2 Estimation of genome features using the k-mer method

The short reads (300–500 bp) from the MGISEQ 2000 platform were quality filtered using HTQC (v1.92.310) (Yang et al., 2013) as described below. First, the adaptors were removed from the sequencing reads. Thereafter, read pairs were excluded if any one end had an average quality lower than 20. The ends of reads were trimmed if the average quality was lower than 20 in the sliding window size of 5 bp. Finally, read pairs with an end shorter than 75 bp were removed. Quality-filtered reads were used for genome size estimation. We generated the 17-mer occurrence distribution of sequencing reads from short libraries using the k-mer method (Guo et al., 2015). The proportion of repeat sequences and the heterozygosity rate of the genome were determined using the GEF (v1.02) (Liu et al., 2013).

### 2.3 Genome assembly using third-generation long reads

We generated 158.95 Gb subreads using one SMRT cell in the PacBio platform by removing adaptor sequences within sequences. The longest 150X subreads data were used for genome assembly of *P. vachelli*. The draft assembly of the genome was assembled using NextDenvo (seed_cutoff = 33348; read_cutoff = 1000) (Zhang J. et al., 2020). The assembly results were corrected by Arrow (v2.10) (Wang et al., 2010) using subreads and PILON (v1.2.2) using the second-generation data (Walker et al., 2014). Haplotigs (default parameter) (Kronenberg et al., 2021) was used to remove heterozygous redundancy after error correction.

### 2.4 Chromosome assembly using Hi-C technology

Muscle from the same sample was used to construct a Hi-C chromatin contact map to enable chromosome-level assembly. To construct the Hi-C library, samples were cross-linked with 1% formaldehyde for 10 min at room temperature and quenched with 0.125 M final concentration glycine for 5 min. The cross-linked cells were subsequently lysed. Endogenous nuclease were inactivated with 0.3% SDS, then chromatin DNA were digested by 100 U MboI (NEB), and marked with biotin-14-dCTP (Invitrogen) and then ligated by 50 U T4 DNA ligase (NEB). After reversing cross-links, the ligated DNA was extracted through QIAamp DNA Mini Kit (Qiagen, Hilden, Germany) according to manufacturers’ instructions. Purified DNA was sheared to 300- to 500-bp fragments and were further blunt-end repaired, A-tailed and adaptor-added, followed by purification through biotin-streptavidin–mediated pull-down and PCR amplification. Finally, the Hi-C libraries were quantified and sequenced on the or MGI-seq platform. The final library was sequenced on the MGISEQ 2000 platform with 150 paired-ends. Thereafter, the clean read pairs from Hi-C library sequencing were mapped to the polished genome using BWA (v0.7.16) (Xie et al., 2022). Paired reads with mate mapped to a different contig were used to perform the Hi-C-associated scaffolding. Self-ligation, non-ligation, and other invalid reads, such as Start NearRsite, PCR amplification, random breaks, large small fragments, and extreme fragments, were filtered. Thereafter, Juicer (v2.0) (Durand et al., 2016b) and a 3D *de novo* assembly (3d-DNA, v170123) (Dudchenko et al., 2017) were used to determine the location and direction of each contig. Finally, JuiceBox (v1.1) (Durand et al., 2016a) was applied to correct the contig orientation and to remove suspicious fragments in the contig to unanchored groups by visual inspection.

### 2.5 Annotation of repetitive sequences

The following methods were combined to identify the repeat contents in the *P. vachelli* genome: homology-based and *de novo* prediction. Homology-based analysis: we identified the known TEs within the *P. vachelli* genome using RepeatMasker (v4.1.0) (Zhang et al., 2022) with the Repbase TE library (Jurka et al., 2005). RepeatProteinMask searches were also conducted using the TE protein database as a query library. *De novo* prediction: we constructed a *de novo* repeat library of the *P. vachelli* genome using RepeatModeler, which automatically executes two core *de novo* repeat-finding programs, namely RECON (v1.08) (Bao and Eddy, 2002) and RepeatScout (v1.0.5) (Price et al., 2005), to comprehensively conduct, refine, and classify consensus models of putative interspersed repeats for the *P. vachelli* genome. We also performed a *de novo* search for long terminal repeat (LTR) retrotransposons against the genome sequences using LTR_FINDER (v1.0.7) (Xu and Wang, 2007). Thereafter, we identified tandem repeats using the Tandem Repeat Finder (TRF) (https://tandem.bu.edu/trf/trf.html), including low-complexity repeats, satellites, and simple repeats. Finally, we merged the library files of the two methods and identify the repeat contents.

### 2.6 Functional annotation of protein-coding genes

We predicted protein-coding genes of the *P. vachelli* genome using three methods, including *ab initio* gene prediction, homology-based gene prediction and RNA-Seq-aided gene prediction. Prior to gene prediction, the assembled genome was hard and soft masked using RepeatMasker (v 4.1.0) (Zhang et al., 2022). We adopted Augustus (v3.2) (Ng et al., 2021) and Genescan (v3.1) (Vidal et al., 2021) to perform *ab initio* gene prediction. Models used for each gene predictor were trained from a set of high-quality proteins generated from homology annotation result. We used Exonerate (v2.2.0) (Chakraborty et al., 2021) to conduct homology-based gene prediction with the default parameters. To carry out RNA-Seq-aided gene prediction, we first mapped the clean RNA-Seq reads into reference sequences using TopHat (v2.1.1) (Trapnell et al., 2009), and the gene structures were built using Cufflinks (v2.2.1) (Hards et al., 2022). Finally, Maker (3.01.03) (Cantarel et al., 2008) was used to integrate the prediction results of the three methods to predict genes modles. The output included a set of consistent and non-overlapping sequence assemblies, which were used to describe the gene structures.

For gene function annotation, we used BLASTP to align the candidate sequences to the Swissport protein databases, TrEMBL and Kyoto Encyclopedia of Genes and Genomes (KEGG) database with an E-value threshold of 1E-5. The protein domains were annotated using PfamScan and InterProScan based on the InterPro protein databases (http://www.ebi.ac.uk/interpro/) (Sidhu et al., 2020). The motifs and domains within the gene models were identified using the PFAM databases (http://pfam.xfam.org/). Gene Ontology (GO) IDs for each gene were obtained from Blast2GO (Conesa et al., 2005).

### 2.7 Gene family identification and phylogenetic analysis

To cluster families from protein-coding genes, proteins from the longest transcripts of each gene from *P. vachelli* and other 16 closely related species (Supplementary Table S6) were extracted and aligned to each other using BLASTp with a maximal E-value of 1E-5. To exclude putative fragmented genes, we filtered out genes with identity less than 30%, coverage less than 50%, and genes encoding protein sequences shorter than 100 bp. The OrthoMCL (v2.0.9) (Li et al., 2003) method was used to cluster genes from these different species into gene families with the parameter of “-inflation 1.5.” The protein sequences of the single-copy ortholog genes were aligned with the MUSCLE (v3.8.31) (Edgar, 2004) program, and the corresponding coding DNA sequence (CDS) alignments were generated and concatenated with the guidance of protein alignment. RAxML (v8.2.11) (Stamatakis, 2014) was used to construct the phylogenetic tree using the maximum likelihood method. Thereafter, the MCMCtree program in the PAML package (v4.8) (Yang, 2007) was used to calculate the divergence time. Eight potential fossil records were downloaded from the TimeTree database (http://www.timetree.org) to calibrate the results.

### 2.8 Gene family expansion and contraction analysis

Based on the identified gene families and the constructed phylogenetic tree with predicted divergence time of those species, we used CAFE4 (Han et al., 2013) to analyze gene family expansion and contraction. A random birth and death model is proposed in CAFE to study gene gain or loss in gene families across a specified phylogenetic tree. Herein, a conditional *p*-value was calculated for each gene family, and families with conditional *p-*value less than 0.05 were considered to have an accelerated rate for gene gain or loss. Such expansion and contraction gene families in *P. vachelli* (*p* < 0.05) were mapped to KEGG pathways for functional enrichment analysis, which was conducted using enrichment methods. Functional enrichment analysis of expansion and contraction gene families was performed against the background of all KEGG-annotated genes in the *P. vachelli* genome. Hypergeometric test algorithms were implemented for the analysis, and the Q-value (FDR, false discovery rate) was calculated to adjust the *p*-value using the R method p. adjust (https://github.com/StoreyLab/qvalue).

### 2.9 Transcriptome sequencing of *P. vachelli* under environmental stress

The experimental fish (19 ± 1.77 g weight, 13 ± 1.36 cm length, 120 days after hatching) were temporarily raised in the circulating water tank (equipped with cooling and heating functions and a volume of 200 L and flow rate of 5 L/min) for 2 weeks, and the formal experiment was carried out after stopping feeding for 2 days. Bacterial infection methods are as follows: To determine the 50% lethal concentration (LC_50_) o f *Edwardsiella ictaluri* (Zhejiang Institute of Freshwater Fisheries, Huzhou, China) at 24 h, a total of 30 fish were intraperitoneally administered with 0.1 ml of 10^3^, 10^4^, 1 0^5^, 10^6^, or 10^7^ CFU/ml (Colony Forming Unit, CFU) of *E. ictaluri*. Fish mortality was monitored every 2 h. The LC_50_ was determined to be 2.0 × 10^4^ CFU/ml. The experimental group was intraperitoneally injected with 0.1 ml *E. ictaluri* at a density of 2.0 × 10^3^ CFU/ml (1/10th LC_50_) and the control group was injected with an equal volume of phosphate buffer solution (PBS, ×1). At 24 h after *E. ictaluri* challenge, liver tissues were quickly dissected, and the samples included three control groups (BC1, BC2, BC3) and three treatment groups (BT1, BT2, BT3). The experimental methods of high temperature stress were as follows: control groups at 25°C, treatment groups at 33°C, after 72 h, the experimental fish were dissected and the liver tissues of the control groups (WC1, WC2, WC3) and the treatment groups (WT1, WT2, WT3) were obtained. The hypoxic transcriptome data was obtained from our previous experiment (Zhang et al., 2016b) with re-annotating based on the *P. vachelli* genome. The specific experimental process is as follows: based on the pre-experiment, we chose 0.7 mg/L as the oxygen concentration level for creating a hypoxic condition. Control fish (P0 a, P0 b, P0 c) were removed from three aquaria for immediate liver dissection. Next, the oxygen infiltration and recirculation systems in the three aquaria were closed to initiate the hypoxia experiments. The water was deoxygenated for 30–35 min by bubbling pure nitrogen gas in order to decrease oxygen concentration from 6.8 to 0.7 mg/L. After oxygen concentration was maintained for 4 h by continuous bubbling of nitrogen gas, the experimental fish (P4 a, P4 b, P4 c) were quickly removed for liver dissection. In all of these experiments, the samplings of control fish and experimental fish had three biological replicates, each made up of three different individual liver tissues.

Total RNA was extracted using Trizol reagent (Invitrogen, CA, United States) following the manufacturer’s instructions. RNA quantity and purity were analyzed with Bioanalyzer 2100 and RNA 6000 Nano LabChip Kit (Agilent, CA, United States) with RIN number >7.0. For cDNA library constructions, Poly(A) RNA was purified from total RNA (5 μg) using poly-T oligo-attached magnetic beads using two rounds of purification. Following purification, the mRNA was fragmented into small pieces using divalent cations under elevated temperature. Then the cleaved RNA fragments were reverse-transcribed to create the final cDNA library in accordance with the protocol for the TruSeq Stranded mRNA Sample Prep Kit (Illumina, San Diego, United States), the average insert size for the paired-end libraries was 300 bp (±50 bp). We then performed the paired-end sequencing on an Illumina Hiseq 4000 (LC Sciences, United States) following the vendor’s recommended protocol. The filtered Clean Data was assembled and annotated based on the *P. vachelli* genome.

### 2.10 Population evolution

#### 2.10.1 Sample collection and genome resequencing

Genome resequencing samples were collected from three populations in the upper (SC), middle (HB), and lower reaches (JS) of the Yangtze River (Figure 3A), with five individuals per population. DNA was extracted from the muscle tissue and subjected to 0.8% agarose gel electrophoresis. The qualified DNA was randomly broken into fragments of 350 bp in length, and the 1 µg DNA per sample was used as the input material and sequencing libraries were generated using the VAHTS Universal DNA Library Prep Kit for MGI (Vazyme, Nanjing, China) following the manufacturer’s. The DNA fragments were repaired by ends. Thereafter, poly A tails followed by sequencing adapters were added. The DNA fragments were purified and amplified by PCR to complete the entire library preparation. After the library was constructed, Qubit 2.0 (Invitrogen Ltd., Paisley, United Kingdom) was used for preliminary quantification, and the library was diluted to 1.5 ng/μl. Then Agilent 2100 BioAnalyzer (California, United States) was used to detect the insert size of the library. qRT-PCR was used to accurately quantify the effective concentration of the library to ensure the quality of the library. After the library quality was verified, the MGISEQ 2000 platform was used for PE150 sequencing. The following four main steps comprise the raw data filtering method: 1) filter out reads containing the adapter sequence; 2) remove leading and trailing bases with base quality less than 20; 3) delete reads less than 50 bp in length; and 4) retain only paired reads.

#### 2.10.2 Variation detection and annotation

Clean reads were compared to the reference genome using BWA (v0.7.16) (Xie et al., 2022), and duplicate reads were processed using Picard software (v1.87) (Kambouris et al., 2014). The mapping rate, coverage, and sequencing depth of the filtered data were analyzed. We further filtered the calls using GATK VariantFiltration (Lin et al., 2022) with the following parameters: QD < 2.0, FS > 60.0, MQ < 40.0, MQRankSum < −12.5, ReadPosRankSum < −8.0. Mutational positions, genomic regions, and potential amino acid changes were assessed using ANNOVAR (v2019). The SNP calls for each sample were then combined using GATK CombineGVCFs under default settings. Each SNP position were further assessed and filtered if: 1) minor allele frequency <0.01, 2) samples with missing genotypes >0.2, and 3) sequencing depth <4.

#### 2.10.3 Population genetic structure analysis

All the SNPs that passed filtering in the population were used to construct a phylogenetic tree using treebest (http://treesoft.sourceforge.net/treebest.shtml) based one Neighbor-Joining Algorithm. The final phylogentic tree was plotted using Itol (https://itol.embl.de). Principal Component analysis was performed using GCTA (v1.91.4) (Yang et al., 2011). The input files for GCTA was converted from VCF files using Plink (v1.9) (Purcell et al., 2007). Admixture (v1.3.0) (Ioannidis et al., 2020) was employed to analyze the population structure based on the Bayesian mathematical model and SNP markers to determine the total population structure. The best K value was determined by calculating the cross-validation error, and finally the optimal number of groups in the population.

#### 2.10.4 Analysis of effective population size and population history dynamics

Based on the SNP markers, the linkage imbalance (LD) between markers can be used to accurately estimate the effective population size. The LD level was evaluated using a pair-wise algorithm, and PopLDdecay (v3.3.1) (Wu et al., 2022) was used to plot the LD decay graph. The demographic history of *P. vachelli* was analysed with the MSMC (v2.0.0) (Schiffels and Wang, 2020). The synonymous mutation rate per base per year was inferred based on formula T = ks/(2λ).

## 3 Results and discussion

### 3.1 Genome sequencing, assembly, and annotation

#### 3.1.1 Genome sequencing and assembly

**FIGURE 1:**
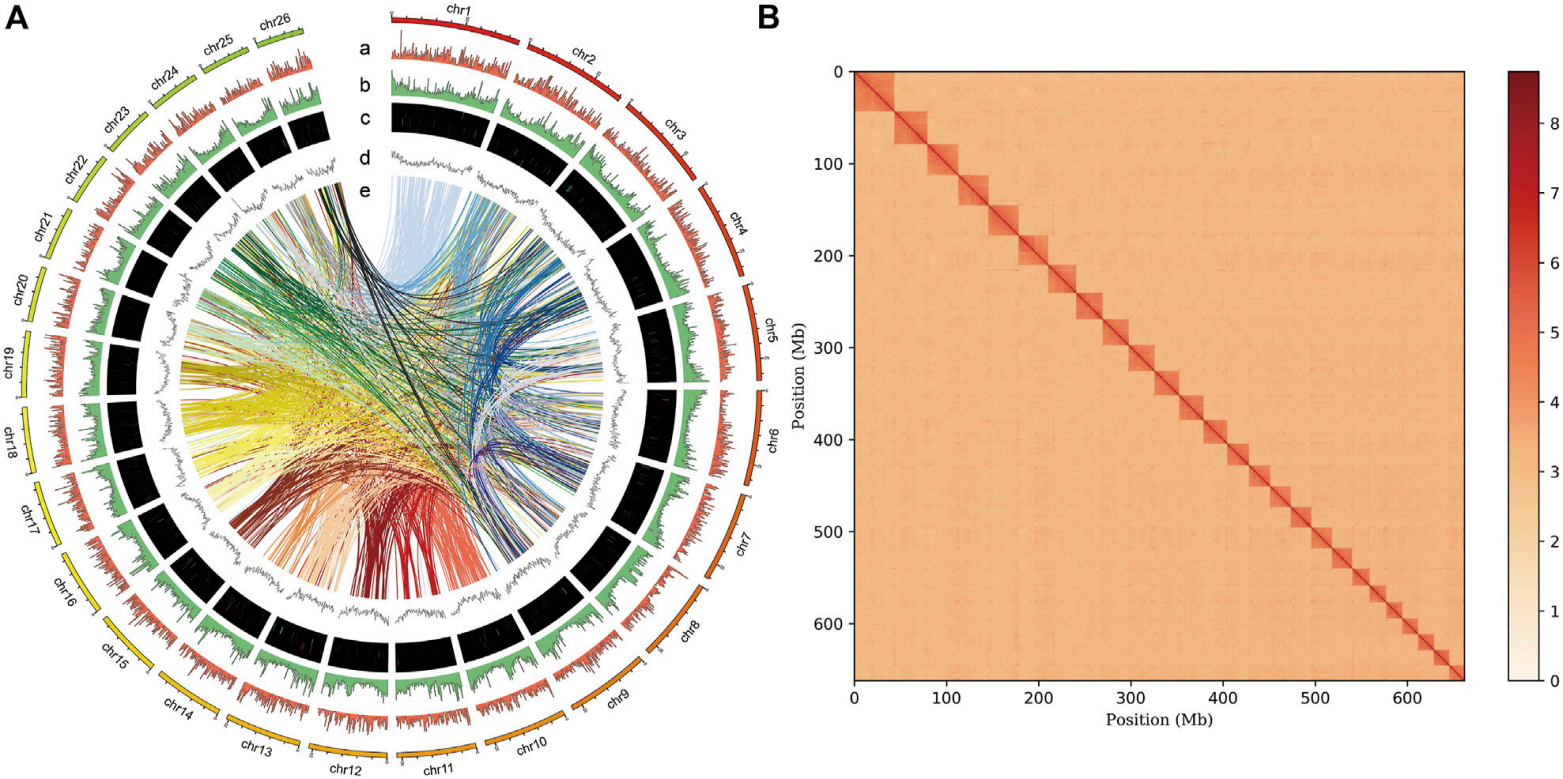
Genome assembly of *P. vachelli*. **(A)** Characteristics of 26 chromosomes of *P. vachelli*. Tracks from the outside to the inside represent distribution of gene density (a), repetitive sequence density (b), distribution of non-codling RNAs (ncRNAs) (c), GC content (d) and gene collinearity (e). Band width is proportional to the syntenic block size. **(B)** Interaction between chromosomes of the *P. vachelli* scaffolds. The blocks represent the contacts between one location and another. The color reflects the intensity of each contact, with deeper colors representing higher intensity.

A total of 80.2 Gb clean reads were produced using the MGISEQ 2000 platform (Table 1). Genome size, heterozygosity, and repeat content of the genome are crucial for designing the strategy for PacBio long-read genome sequencing and assembly. Based on short read data and 17-mer analysis (Supplementary Figure S1), we estimated a genome size of 675.72 Mb, a heterozygosity rate of 0.45%, and repeat ratio of 43.31%. Thereafter, ∼158 Gb subreads data were produced for reference genome construction using the PacBio platform (Table 1). After assembly based on subreads data, a ∼663.50 Mb high-quality genome was obtained, with a contig N50 of 16.67 Mb. We also obtained ∼69 Gb Hi-C clean reads, and genome assembly spanned 662.13 Mb with a contig N50 = 14.02 Mb (Table 2) and GC content of 39.5% (Supplementary Figure S2), thereby accounting for 99.79% of the original genome length. Based on Hi-C scaffolding, we anchored the genome to 26 chromosomes (Figure 1B, Supplementary Table S1), which was congruent with the *P. vachelli* karyotype (2n = 52) (Zhang et al., 2020a). Finally, the assembled genome was evaluated by BUSCO pipeline, the assembly contained 92.7% complete and 1.4% fragmented conserved single-copy ortholog genes (Supplementary Table S2). Such finding indicates that the genome assembly has high coverage and completeness.

**TABLE 1:** Statistics of the genome sequencing data.

Libraries	Data type	Reads number	Base count (bp)	Average read length (bp)	Experiment insert size (bp)
Pacbio reads	Clean data	10164887	158,946,540,720	15636.82	40960
NGS reads	Clean data	534,573,526	80,186,028,900	150	300–500
Hi-C reads	Clean data	461,481,580	68,668,773,806	148	300–500

**TABLE 2:** Statistics of the *P. vachelli* genome before and after chromosome level assembly.

	Primary genome assembly	Chromosome-level genome assembly
Total (bp)	663,496,518	662,130,887
Contig Number	93	101
Contigs N50 (bp)	16,665,156	14,023,858
Scaffold Number	—	26
Scaffold N50 (bp)	—	26,782,657

#### 3.1.2 Genome annotation

A total of 231.60 Mb of repetitive elements were identified, accounting for 34.90% of the *P. vachelli* genome (Figure 1A). Notably, this value was lower than that of *Lethenteron reissneri* (57.25%), *Epinephelus lanceolatus* (41.01%), and *Danio rerio* (52.50%), and markedly higher than that of *Gasterosteus aculeatus* (25.20%), *Larimichthys crocea* (18.1%), and *Oryzias latipes* (17.5%). The most abundant TEs were DNA transposons (19.13%), followed by long interspersed elements (LINEs, 8.63%) and long terminal repeats (LTRs, 7.53%) (Supplementary Table S3).

A protein-coding gene set comprising 21,974 genes was predicted by integrating *de novo* and homology searching methods (Supplementary Table S4). Approximately 97.87% of the protein-coding genes exhibited a significant sequence-level similarity to entries of other species using at least one public database, the quality of which was comparable to that of published high-quality gene sets (Supplementary Table S5). By comparing the distribution of genes, coding sequences (CDS), exon and intron lengths, exon and intron numbers, and the gene and CDS gene content of *P. vachelli* to those of closely related species, we found that their distributions in the *P. vachelli* genome were comparable to those of other teleosts (Supplementary Figure S3). Furthermore, 91.7% complete BUSCO genes were successfully identified (Supplementary Table S2). The above results show that our genome annotation is of high integrity and quality for further analysis.

### 3.2 Comparative genomics analysis

**FIGURE 2:**
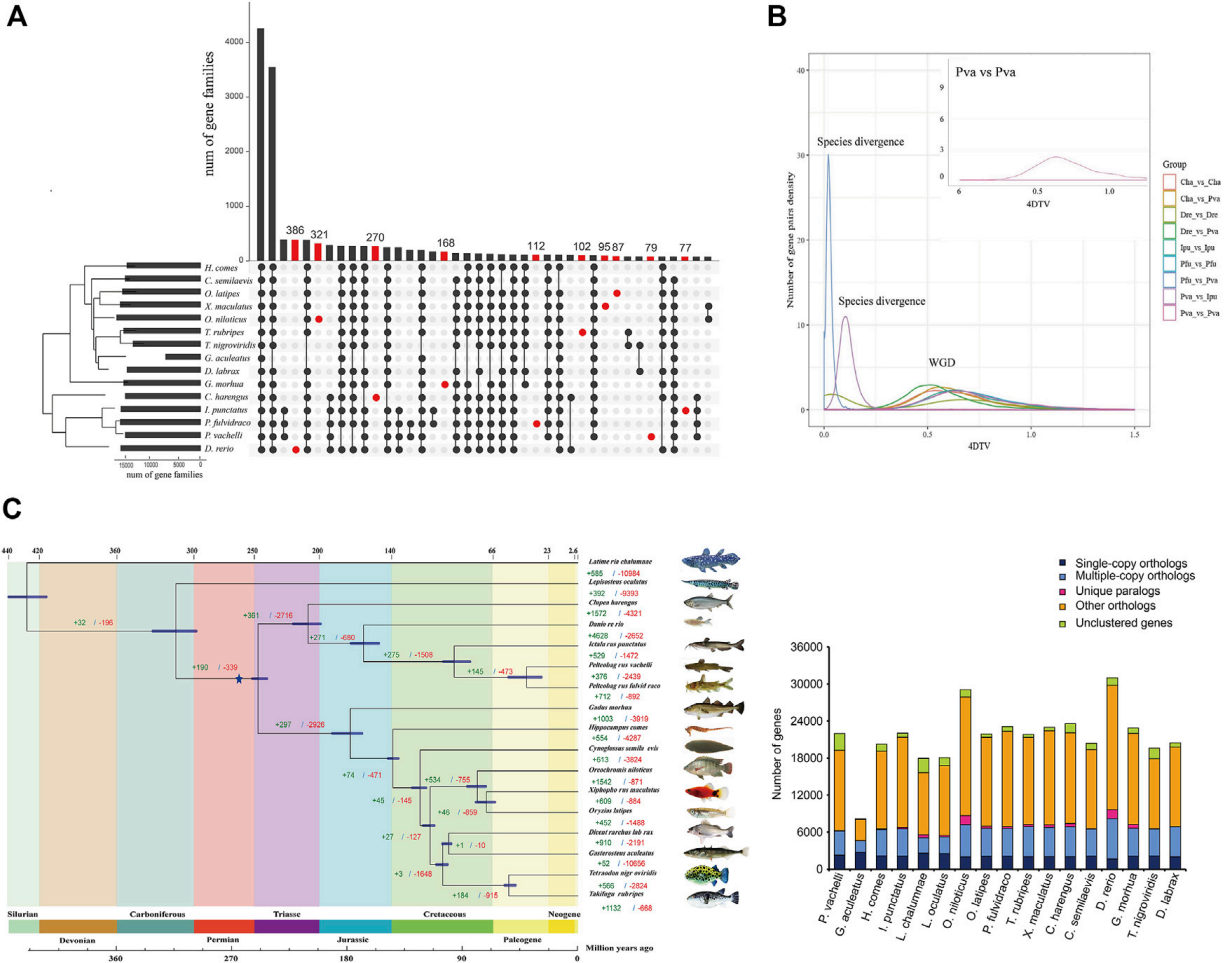
Comparative genomics analysis of *P. vachelli*. **(A)** An UpSet plot of gene families in *P. vachelli*a and other fishes. The UpSet Plot was drawn using the R package ‘UpSet’. The abscissa of the horizontal bar chart represents the total number of gene families in each species, the ordinate represents the size of gene families, the red dots represent unique gene families, and the line segment represents the intersection relationship. **(B)** 4DTv prediction of the timing of WGD. Different colors indicate the comparisons between different species. Cha = *Clupea harengus*, Dre = *Danio rerio*, Ipu = *Ictalurus punctatus,* Pfu = *Pelteobagrus fulvidraco,* Pva = *Pelteobagrus vachelli*. **(C)** Inferred phylogenetic tree with 1,106 single-copy orthologs of 17 species identified by RAxML. Blue star represented whole-genome duplication (WGD) events. The green numbers represent the expansion gene familys and red numbers represent the contraction gene familys.

To investigate the phylogenetic relationship of *P. vachelli* with other species, we compared the genomes of *P. vachelli* and other published vertebrate species (Figure 2C). Gene family UpSet plot showed the interactive genes between *P. vachelli* and its relatives fish species, the number of gene families shared by all species was the largest, and *P. vachelli* had 79 unique gene families (Figure 2A). Further, we performed an intergenomic co-linearity analysis of *P. vachelli* and its closest relatives, and the results showed the homology of the genomes of *P. vachelli, P. fulvidraco,* and *I. punctatus* (Supplementary Figure S4). Additionally, a phylogenetic tree based on single-copy orthologs was constructed (Figure 2C), in which the divergence time of *P. fulvidraco* and *I. punctatus* (96.4 Mya) were similar to those presented in other reports (Gong et al., 2018)*.* The phylogenetic tree showed that the divergence time between *Pelteobagrus* and *Ictalurus* was approximately 166.9 (155.0–176.6) million years ago (Mya). In addition, the *P. fulvidraco* was the most closely related to *P. vachelli* with a divergence time around 40.0 (28.5–54.0) Mya.

Whole-genome duplication (WGD) events are an important driving force for species evolution (Walden et al., 2020). Ray-finned fish evolution spanned more than 400 million years (Near et al., 2012). In addition to the two rounds of WGD that occurred at the root of the vertebrate lineage (VGD1 and VGD2, 320–350 Mya) (Dehal and Boore, 2005), teleost fish experienced a third round of WGD (TSGD) (Bayes et al., 2017). Additional WGDs have also been described in the teleost lineage including the salmonid-specific WGD (SaGD) that occurred about 100 Mya (Larhammar and Risinger, 1994). Previous studies have shown that the peak around 0.6 in 4DTv represents the TSGD, such as zebrafish and carp (4DTv = 0.58) (Xu et al., 2014). In this study, the 4DTv results showed a peak around 0.6 (Figure 2B), which was consistent with the 4DTv of TSGD. Furthermore, the WGD (Figure 2C) in this study (around 270 Mya) was consistent with the TSGD time (226–316 Mya) (Pasquier et al., 2016) reported in previous studies, indicating the WGD belongs to TGSD.

### 3.3 Analysis of environmental adaptation evolution in *P. vachelli*

#### 3.3.1 Gene family comparison

**FIGURE 3:**
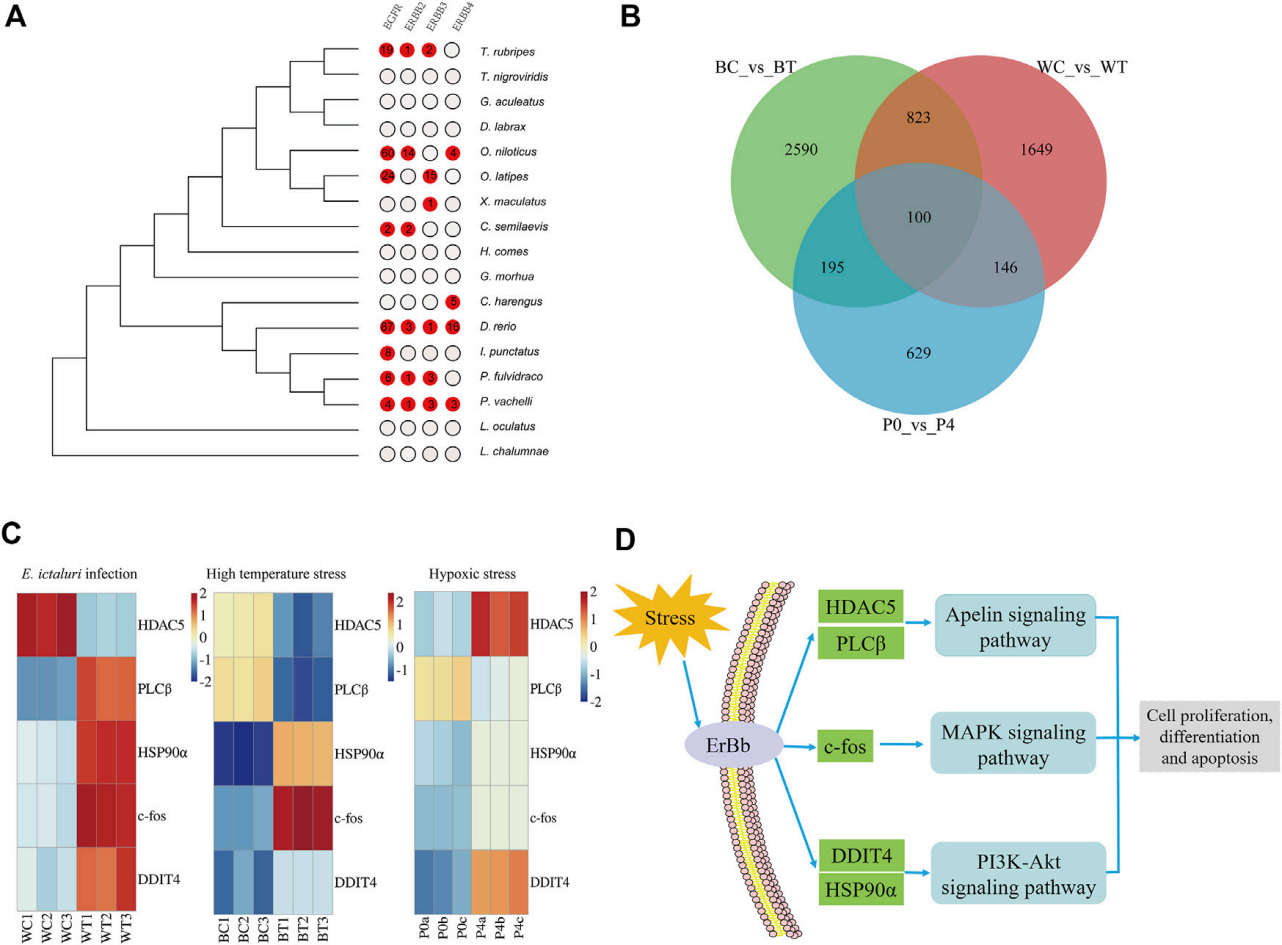
Evolutionary analysis of environmental adaptation in *P. vachelli*. **(A)** Evolution of the ErbB family in *P. vachelli* and other fishes. **(B)** Veen diagram of significantly differentially expressed genes in all transcriptomes. BC vs. BT represent control vs. treatment groups in *E. ictaluri* infection transcriptome, WC vs. WT represent control vs. treatment groups in high temperature stress transcriptome, P0 vs. P4 represent control vs. treatment groups in hypoxic stress transcriptome. **(C)** Transcriptome expression of candidate genes for environmental adaptation evolution in *P. vachelli*. Color gradients represent gene expression level of control group and treatment group. **(D)** Candidate genes and pathways for environmental adaptation evolution in *P. vachelli.*

The expansion and contraction of gene families may be one of the most important factors for phenotypic diversity and evolutionary adaptation to the environment (Harris and Hofmann, 2015). Based on the phylogenetic tree, a gene family comparison analysis was performed, and 386 expanded and 245 contracted gene families were identified in *P. vachelli* (Supplementary Table S7). In the expanded gene families, KEGG enrichment showed that environmental information processing related pathways were significantly enriched (*p* < 0.05), such as ErbB signaling pathway, MAPK signaling pathway, PI3K-Akt signaling pathway and apelin signaling pathway (Supplementary Table S7). In addition, ErbB (receptor tyrosine-protein kinase erbB) family (EGFR, ErbB2, ErbB3, ErbB4) were repeatedly enriched in these pathways. Further, we performed an evolutionary analysis of the ErbB family in *P. vachelli* and other fishes according to the expansion gene family (Figure 3A). The results showed that only the *P. vachelli* and *D. rerio* had completed ErbB family in the evolution process, while other species had different degrees of deletion. There were also differences in gene copy number across species, which might be due to differences in the pressures they face to survive.

#### 3.3.2 Transcriptome analysis of environmental stress

We performed transcriptome sequencing of *P. vachelli* under bacterial infection, hypoxia and high temperature stress respectively (Supplementary Figure S5). In bacterial infection transcriptome, a total of 18,629 unique genes were identified from six complementary DNA libraries (BC1, BC2, BC3, BT1, BT2, BT3), including 3,708 differentially expressed genes (DEGs) (foldchange > 2, *p* < 0.05). KEGG analysis showed that 19 pathways were significantly enriched (FDR <0.05), which were mainly involved in immunity and disease, proteolysis and synthesis, and metabolism (Supplementary Table S8). In hypoxia stress transcriptome, 18,634 unique genes were identified from six DNA libraries (P0a, P0b, P0c, P4a, P4b, P4c), including 1,070 DEGs (foldchange >2, *p* < 0.05). KEGG analysis showed that 14 pathways were significantly enriched (FDR <0.05), which were mainly involved in carbohydrate metabolism, endocrine system, endocrine and metabolic disease (Supplementary Table S8). Notably, the HIF-1 signaling pathway, a key pathway in the hypoxia response, was also significantly enriched (Li et al., 2022). In high temperature stress transcriptome, 18,832 unique genes were identified from six DNA libraries (WC1, WC2, WC3, WT1, WT2, WT3), including 2,718 DEGs (foldchange >2, *p* < 0.05). KEGG analysis showed that 14 pathways were significantly enriched (FDR < 0.05), which were mainly involved in carbohydrate metabolism, immunity and disease, proteolysis and synthesis (Supplementary Table S8). In conclusion, based on the KEGG analysis of DEGs, we found that the response pathways of *P. vachelli* under different environmental stresses was specific. The response pathways under bacterial infection and high temperature stress were similar (mainly involved in immunity and diseases, proteolysis and synthesis), while endocrine related pathways under hypoxia stress may played a role in hypoxia response. Endocrine is an important and tightly regulated system for maintaining body homeostasis. Current studies have shown that hypoxia can induce the dysfunction of endocrine organs, resulting in dysregulation of body homeostasis.

To further explore candidate genes for environmental adaptation, we conducted a venn diagram analysis based on the DEGs of the three transcriptomes, the results showed that they shared 100 genes. (Figure 3B, Supplementary Table S8). Further analysis revealed that among the 100 genes, only five genes [histone deacetylase 5 (HDAC5), phosphatidylinositol phospholipase C beta (PLCβ), proto-oncogene c-Fos (c-fos), DNA damage-inducible transcript 4 protein-like (DDIT4), heat shock protein HSP 90-alpha (HSP90α)] were functional genes in the key KEGG pathway of above expanded gene families (Figure 3C). HDAC5 and PLCβ were involved in apelin signaling pathway, c-fos was involved in MAPK signaling pathway, DDIT4 and HSP90α were involved in PI3K-Akt signaling pathway. According to the KEGG database, it was worth noting that these pathways were interconnected by the ErbB family and involved in the regulation of cell proliferation, differentiation and apoptosis (Figure 3D) (Yarden and Sliwkowski, 2001; Liu et al., 2015; Zhang P et al., 2020), indicating these physiological processes play a role in environmental adaptation of *P. vachelli.* In addition, these five genes had different expression patterns under bacterial infection, hypoxia and high temperature stress, suggesting that unique adaptive mechanisms to different kinds of environmental stress in *P. vachelli* (Figure 3C)*.*

### 3.4 Evolution analysis of the geographical population of *P. vachelli*

**FIGURE 4:**
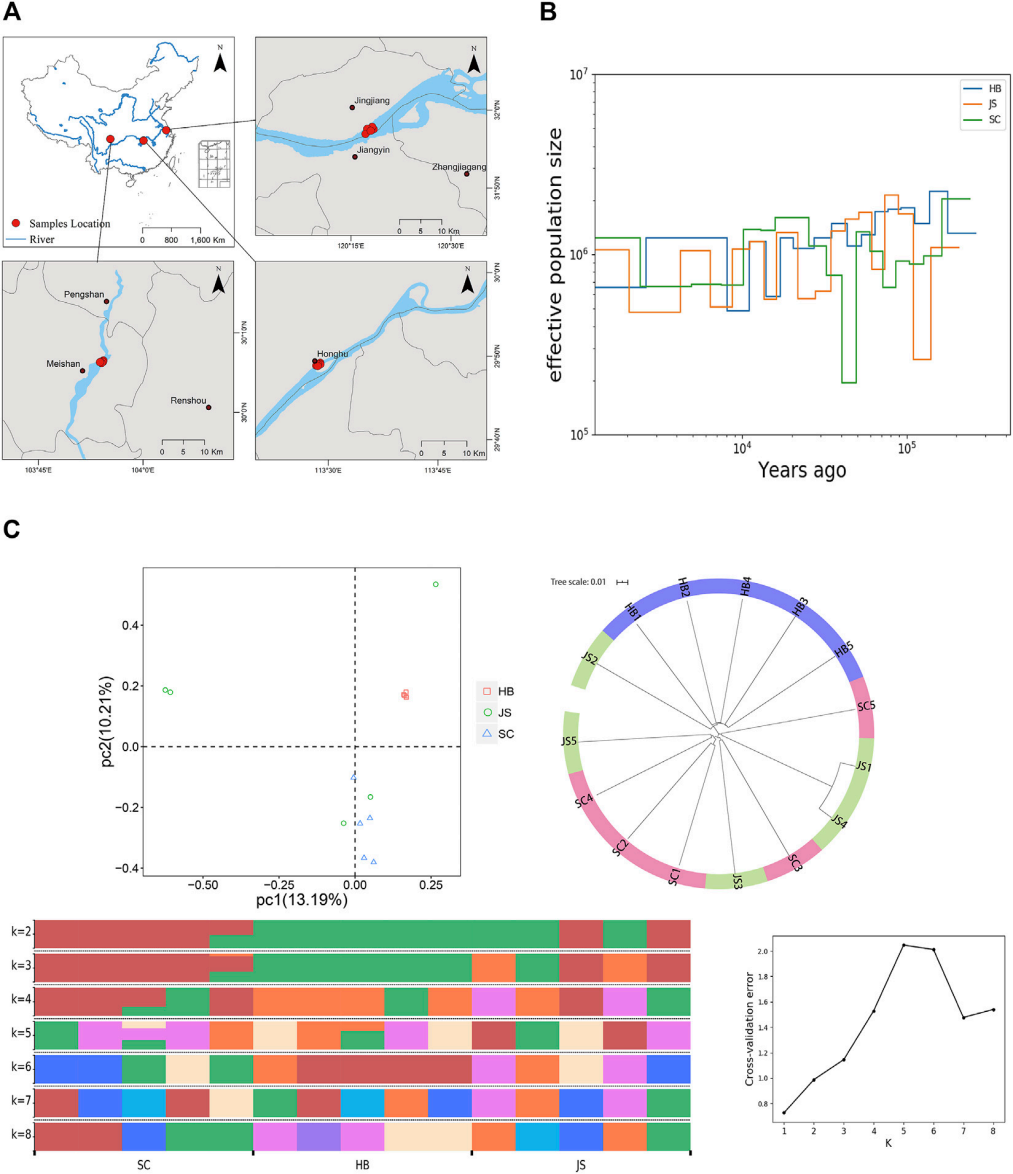
Population evolution of *P. vachelli*. **(A)** Geographical distribution of *P. vachelli*. **(B)** Demographic histories of *P. vachelli*. The blue line represents Hubei *P. vachelli* population (HB), the orange line represents Jiangsu *P. vachelli* population (JS), and the green line represents Sichuan *P. vachelli* population (SC). **(C)** Population structure of *P. vachelli*, including principal component analysis (PCA), phylogenetic tree and genetic structure analysis (different colors indicate different genetic information).

Population evolution research is used to analyze population genetic diversity, genetic structure, gene exchange, species formation mechanism, population evolution dynamics, and other biological issues by obtaining SNP variation information for each subgroup of natural population (Gui et al., 2020; Nematollahi et al., 2021). Based on the natural geographical distribution characteristics of *P. vachelli,* we conducted population evolution analysis of the populations in the upper reaches (SC), middle reaches (HB), and lower reaches (JS) of the Yangtze River (Figure 4A). Based on the analysis of effective population size and population history dynamics, during the evolution process, a difference was found in the effective population size of different geographic populations of *P. vachelli* (Figure 4B, Supplementary Figure S6). The population size of JS and SC declined sharply in 100–200 and 40–50 thousand years ago, respectively, while that of HB did not fluctuate significantly in the evolutionary process. The selection pressure caused by the change of natural environment is an important reason affecting the population size. According to relevant information, a sea level rise event occurred in the lower reaches of the Yangtze River approximately 130 thousand years ago (Otto-Bliesner et al., 2006). We speculate that the rising sea level disrupted the habitat of *P. vachelli* in the lower reaches of the Yangtze River, leading to a significant reduction in its population size.

Principal component analysis (PCA) showed that all HB individuals were clustered together, where SC groups and part of JS individuals clustered together (Figure 4C). In addition, the phylogenetic tree analysis also showed that the SC population and JS population had a close genetic relationship (Figure 4C). Furthermore, we conducted population genetic structure analysis (best K value = 5), and the results showed that JS population was mainly composed of SC and HB population, which was especially obvious when K = 3 or K = 2 (Figure 4C). In conclusion, the genetic relationship among the *P. vachelli* populations contradicted their natural geographical distribution, populations in the upper (SC) and lower reaches (JS) of the Yangtze River showed closer genetic relationship. In recent years, the breeding parents and released fry of *P. vachelli* in the lower reaches of the Yangtze River are mainly sourced from Sichuan, China. Therefore, we speculated that the artificial intervention had affected the *P. vachelli* population genetic structure in the Yangtze River.

## 4 Conclusion

In this study, we established the first high-quality reference genome for *P. vachelli*, which can serve as a basic database for the protection of its germplasm resources. Based on the 4DTv analysis, we found a WGD at about 270 Mya, which was consistent with the reported TSGD time. The MAPK signaling pathway, PI3K-Akt signaling pathway and apelin signaling pathway play an important role in the evolution of environmental adaptation in *P. vachelli,* which were interconnected by the ErbB family and involved in the regulation of cell proliferation, differentiation and apoptosis. Population evolution analysis showed that artificial interventions have affected *P. vachelli* wild populations.

## Data Availability

The datasets presented in this study can be found in online repositories. The names of the repository/repositories and accession number(s) can be found in the article/Supplementary Material.
