# Supplementary material for: A high-quality chromosome-level genome assembly of Pelteobagrus vachelli provides insights into its environmental adaptation and population history
Source: Front Genet. 2022 Nov 14;13:1050192. doi: 10.3389/fgene.2022.1050192 (PMC9702082; doi:10.3389/fgene.2022.1050192)
Supplement: Supplementary file 2 [file DataSheet1.docx]

***Supplementary Materials for***

**A high-quality chromosome-level genome assembly of** ***Pelteobagrus vachelli* provides insights into its environmental adaptation and population history**

Jie Li^#,1,2^, Tao Wang^#,^*^,1,2^, Wei Liu^#,3^, Danqing Yin^4^, Zhengqing Lai^1^,Guosong Zhang^1^, Kai Zhang^1,2^, Jie Ji^1,2^, Shaowu Yin*^,1,2^

# These authors contributed equally to this work.

*Corresponding authors: Tao Wang, E-mails: [seawater88@126.com](mailto:seawater88@126.com); Shaowu Yin, E-mails: yinshaowu@163.com

1. *College of Marine Science and Engineering,* *Jiangsu Province Engineering Research Center for Aquatic Animals Breeding and Green Efficient Aquacultural Technology*, *Nanjing Normal University, Nanjing, Jiangsu 210023, China.*
2. *Co-Innovation Center for Marine Bio-Industry Technology of Jiangsu Province, Lianyungang, Jiangsu 222005, China.*
3. *Institute of Fisheries Science of Nanjing，Nanjing 210029，China.*
4. *School of Biomedical Sciences, Li Ka Shing Faculty of Medicine, University of Hong Kong, Pokfulam, Hong Kong SAR, China*


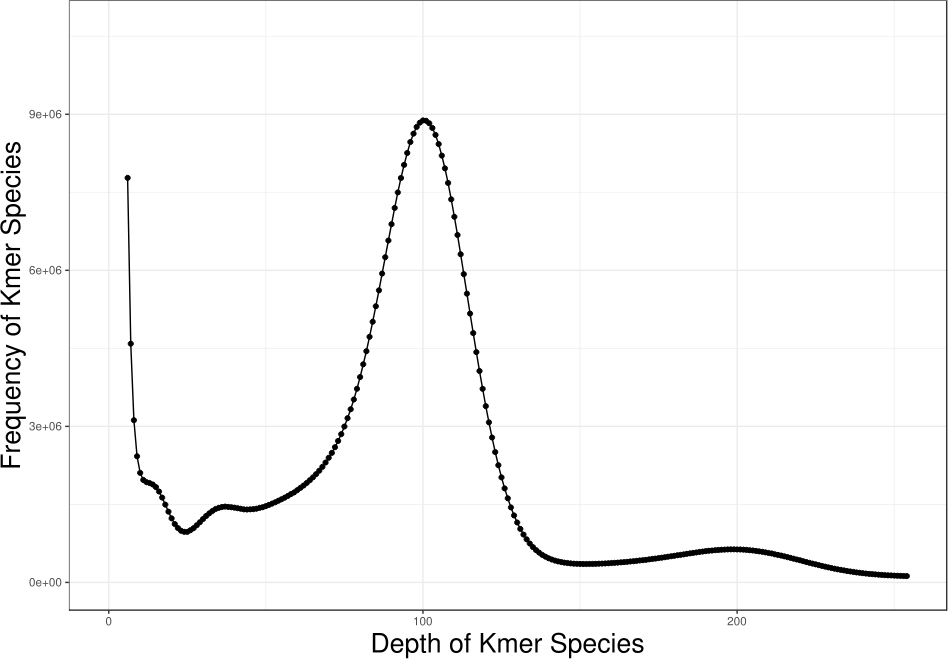


Fig. S1 Genome survey of *P. vachelli* using 17-mer analysis


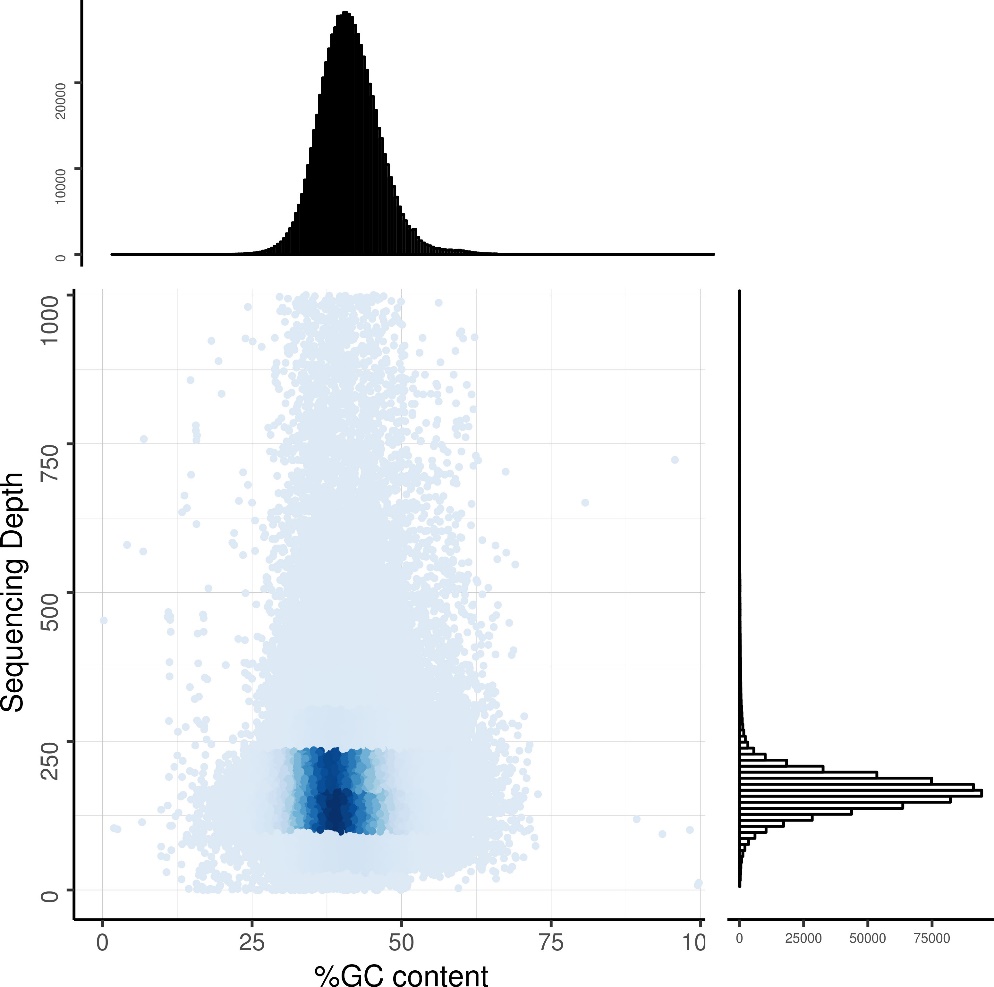


Fig. S2 Sequence depth and GC content of the *P. vachelli* genome .

**
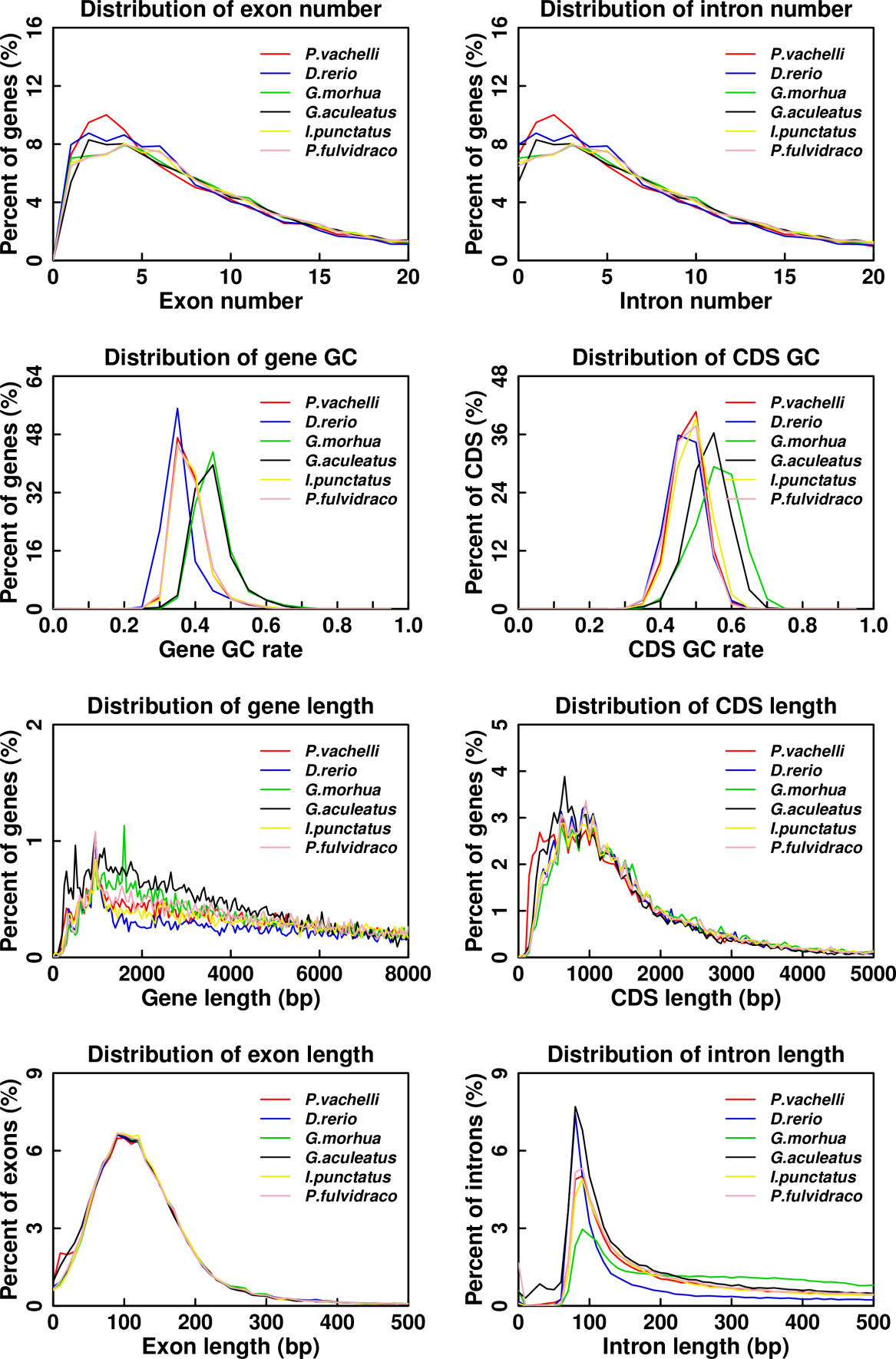
**

Fig. S3 Characteristics comparison of protein-coding genes in genomes of *P. vachelli* and other teleosts.

Fig. S4 Synteny analysis among the genomes *Pelteobagrus vachelli*, *P. fulvidraco* and *Ictalurus punctatus.* (A) Macrosynteny between *P. vachelli*, *P. fulvidraco* and *I. punctatus*. (B) Comparison with P*. fulvidraco* (PF) and *P. vachelli* (PV) chromosomes reveals synteny, the syntenic PF blocks are painted onto PV chromosomes.

Fig. S5 Hierarchical clustering of differentially expressed genes among six libraries. (A) *Edwardsiella ictaluri* infection transcriptome (3 control group BC vs 3 treatment group BT). (B) Hypoxia stress transcriptome (3 control group P0 vs 3 treatment group P4). (C) High temperature stress transcriptome (3 control group WC vs 3 treatment group WT).


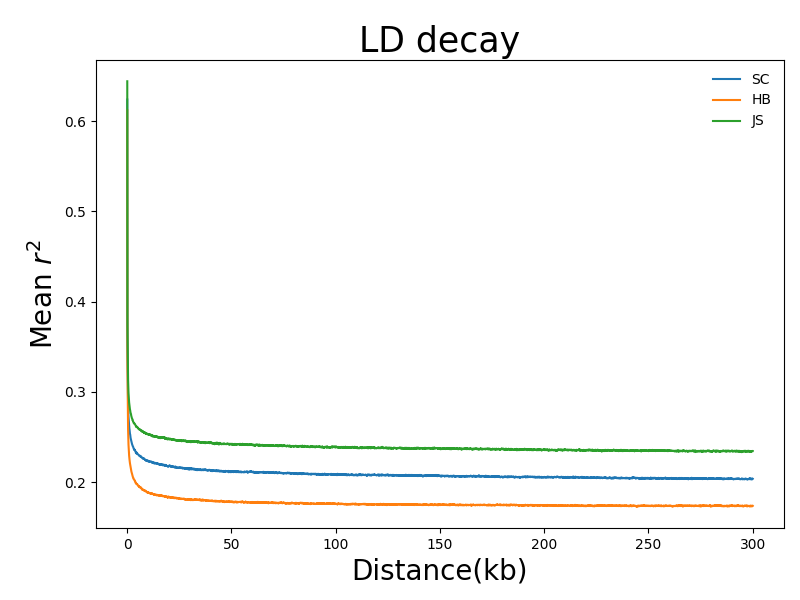


Fig.S6 Linkage disequilibrium (LD) decay of *Pelteobagrus vachelli* geographic populations.

Table S1 Chromosome lengths of the *P. vachelli*

| Superscaffold | Number of Contigs | Length of Contigs (bp) | Length of Superscaffold (bp) |
| --- | --- | --- | --- |
| Superscaffold1 | 7 | 43,552,793 | 43,555,793 |
| Superscaffold2 | 6 | 35,571,735 | 35,574,235 |
| Superscaffold3 | 2 | 33,723,873 | 33,724,373 |
| Superscaffold4 | 3 | 32,900,106 | 32,901,106 |
| Superscaffold5 | 6 | 32,416,300 | 32,418,800 |
| Superscaffold6 | 4 | 32,163,036 | 32,164,536 |
| Superscaffold7 | 4 | 30,600,000 | 30,601,500 |
| Superscaffold8 | 4 | 28,411,188 | 28,412,688 |
| Superscaffold9 | 3 | 28,350,000 | 28,351,000 |
| Superscaffold10 | 2 | 28,145,298 | 28,145,798 |
| Superscaffold11 | 6 | 26,780,157 | 26,782,657 |
| Superscaffold12 | 1 | 26,348,281 | 26,348,281 |
| Superscaffold13 | 11 | 25,896,723 | 25,901,723 |
| Superscaffold14 | 5 | 23,636,113 | 23,638,113 |
| Superscaffold15 | 1 | 22,908,928 | 22,908,928 |
| Superscaffold16 | 2 | 22,358,250 | 22,358,750 |
| Superscaffold17 | 3 | 22,311,938 | 22,312,938 |
| Superscaffold18 | 3 | 22,250,000 | 22,251,000 |
| Superscaffold19 | 2 | 22,249,745 | 22,250,245 |
| Superscaffold20 | 3 | 18,725,326 | 18,726,326 |
| Superscaffold21 | 5 | 17,967,260 | 17,969,260 |
| Superscaffold22 | 1 | 17,647,768 | 17,647,768 |
| Superscaffold23 | 6 | 17,201,210 | 17,203,710 |
| Superscaffold24 | 2 | 17,155,006 | 17,155,506 |
| Superscaffold25 | 4 | 16,673,453 | 16,674,953 |
| Superscaffold26 | 5 | 16,186,400 | 16,188,400 |
| TOTAL | 101 | 662,130,887 | 662,168,387 |

Table S2. BUSCO assessment of the *P. vachelli* genome assembly and annotation

|  | Assembly | | Annotation | |
| --- | --- | --- | --- | --- |
| Term | Proteins | Percentage (%) | Proteins | Percentage (%) |
| complete BUSCOs | 4,250 | 92.7 | 4200 | 91.7 |
| Complete and single-copy BUSCOs | 4,071 | 88.8 | 3940 | 86.0 |
| Complete and duplicated BUSCOs | 179 | 3.9 | 260 | 5.7 |
| Fragmented BUSCOs | 63 | 1.4 | 106 | 2.3 |
| Missing BUSCOs | 271 | 5.9 | 278 | 6.0 |
| Total BUSCO groups searched | 4,584 | 100.0 | 4585 | 100 |

Table S3. Categorization of repetitive sequences

| Type | Repeat Masker TEs  (Repbase+Denovo) | | Repeat Protein Mask TEs | | Combined TEs | |
| --- | --- | --- | --- | --- | --- | --- |
|  | Length(bp) | %in Genome | Length (bp) | % in Genome | Length(bp) | % in Genome |
| DNA | 76,377,513 | 11.51 | 232,667 | 0.04 | 126,941,218 | 19.13 |
| LINE | 25,491,478 | 3.84 | 16,800,962 | 2.53 | 57,253,770 | 8.63 |
| SINE | 15,732,540 | 2.37 | 0 | 0.00 | 21,308,179 | 3.21 |
| LTR | 15,528,438 | 2.34 | 6,861,644 | 1.03 | 49,980,860 | 7.53 |
| Other | 13,409,564 | 2.02 |  |  | 23,644,032 | 3.56 |
| Unknown | 456,025 | 0.07 | 0 | 0.00 | 1,485,802 | 0.22 |
| Total | 132,113,227 | 19.91 | 23,885,345 | 3.60 | 231,599,803 | 34.90 |

Table S4. Statistics of gene prediction

| Gene set | | Number | Average gene  length(bp) | Average CDS  length(bp) | Average exon per length(bp) | Average exon  length(bp) | Average intron  length(bp) |
| --- | --- | --- | --- | --- | --- | --- | --- |
| *De novo* | AUGUSTUS | 17,603 | 16,245.98 | 1,581.11 | 9.12 | 173.33 | 1,805.55 |
|  | Genscan | 23,710 | 19,141.00 | 1,549.39 | 8.26 | 187.64 | 2,423.94 |
| Homolog | *G.morhua* | 43,285 | 8,087.48 | 879.66 | 4.80 | 183.11 | 1,894.84 |
|  | *G.aculeatus* | 32,352 | 8,702.21 | 979.56 | 5.73 | 170.91 | 1,632.24 |
|  | *I.punctatus* | 45,763 | 8,606.38 | 973.61 | 5.26 | 185.14 | 1,792.29 |
|  | *P.fulvidraco* | 52,959 | 6,961.34 | 864.42 | 4.60 | 188.07 | 1,695.34 |
|  | *D.rerio* | 44,363 | 8,647.72 | 1,003.06 | 5.08 | 197.62 | 1,875.73 |
| BUSCO | | 4,533 | 15,043.30 | 1,977.62 | 13.89 | 142.34 | 1,013.34 |
| MAKER | | 23,510 | 13,995.67 | 1,353.44 | 8.55 | 221.35 | 1,602.68 |
| HiCESAP | | 21,974 | 14,636.47 | 1,570.73 | 9.50 | 202.61 | 1,495.69 |

Table S5 Gene annotation in different databases

| Type | Number | Percent (%) |
| --- | --- | --- |
| InterPro | 18,962 | 86.29 |
| GO | 14,483 | 65.91 |
| KEGG_ALL | 21,335 | 97.09 |
| KEGG_KO | 13,760 | 62.62 |
| Swissprot | 19,917 | 90.64 |
| TrEMBL | 21,123 | 96.13 |
| NR | 20,995 | 95.54 |
| Annotated | 21,506 | 97.87 |
| Total | 21,974 | - |

Table S6 Public teleost genomics for comparative genomics analysis

| Species | Genebank no. |
| --- | --- |
| *Gasterosteus aculeatus* | GCA_006229165.1 |
| *Hippocampus comes* | GCF_001891065.1 |
| *Ictalurus punctatus* | GCF_001660625.1 |
| *Latimeria chalumnae* | GCF_000225785.1 |
| *Lepisosteus oculatus* | GCF_000242695.1 |
| *Oreochromis niloticus* | GCF_001858045.2 |
| *Oryzias latipes* | GCF_002234675.1 |
| *Pelteobagrus fulvidraco* | GCF_003724035.1 |
| *Takifugu rubripes* | GCF_901000725.2 |
| *Xiphophorus maculatus* | GCF_002775205.1 |
| *Clupea harengus* | GCF_900700415.1 |
| *Cynoglossus semilaevis* | GCF_000523025.1 |
| *Danio rerio* | GCF_000002035.6 |
| *Gadus morhua* | GCF_902167405.1 |
| *Tetraodon nigroviridis* | GCA_000180735.1 |
| *Dicentrarchus labrax* | GCA_000689215.1 |
